# Plasma boron concentrations in the general population: a cross-sectional analysis of cardio-metabolic and dietary correlates

**DOI:** 10.1007/s00394-021-02730-w

**Published:** 2021-11-26

**Authors:** Katharina S. Weber, Ilka Ratjen, Janna Enderle, Ulrike Seidel, Gerald Rimbach, Wolfgang Lieb

**Affiliations:** 1 Institute of Epidemiology, Kiel University, Niemannsweg 11, 24105 Kiel, Germany; 2 Institute of Human Nutrition and Food Science, Kiel University, 24118 Kiel, Germany

**Keywords:** Trace mineral, PopGen, General population sample, Reduced rank regression, Plant-based diet index, Stepwise forward selection procedure

## Abstract

**Purpose:**

Experimental evidence suggests positive effects of boron on health and metabolism, but human data are still scarce. We aimed to identify dietary and cardio-metabolic correlates of plasma boron concentrations in the general population.

**Methods:**

In a community-based sample (*n* = 899, 57% men, mean age 61 years), plasma boron (median [IQR]: 33.80 µg/L [25.61; 44.65]) concentrations were measured by inductively coupled plasma-mass spectrometry. Overall (PDI), healthy (hPDI), and unhealthy (uPDI) plant-based diet indices were derived from a validated food frequency questionnaire. Reduced rank regression (RRR) yielded a dietary pattern explaining 30% of the variation of circulating boron. Cross-sectional associations of dietary indices and cardio-metabolic traits with plasma boron concentrations were assessed using multivariable-adjusted linear regression analysis.

**Results:**

The RRR pattern was characterized by high intake of fruits, nuts/seeds, tea, wine and low intake of e.g. bread, poultry, processed meat, chocolate/sweets, and soft drinks. 10-point increments in PDI, hPDI, and uPDI were associated with 8.7% (95% CI: 4.2; 13.4), 10.4% (95% CI: 6.6; 14.3), and −8.8% (95% CI: −12.1; −5.4) change in plasma boron concentrations, respectively. Age and phosphate were directly, while BMI, plasma lipid concentrations, and CRP were inversely associated with circulating boron. Plasma boron concentrations were higher in summer vs. winter, in individuals taking vs. not taking antihypertensive medication, and in individuals with high or medium vs. low education level.

**Conclusion:**

Higher plasma boron concentrations appeared to associate with a healthier diet, were related to lower BMI and a more favorable cardio-metabolic risk profile, and showed seasonal variations.

**Supplementary Information:**

The online version contains supplementary material available at 10.1007/s00394-021-02730-w.

## Introduction

Boron is a trace element, which is widely distributed in nature and mainly occurs in the form of borates or organoboron in soil, rocks, and water [1, 2]. Human exposure to boron primarily occurs through oral intake of drinking water and food [1, 3–5], with a boron-rich diet being characterized by high consumption of fruits, vegetables (leafy), nuts, legumes, and plant-derived fermented beverages (i.e. wine, cider, beer) [3, 4, 6]. Poor sources of boron are meat, fish, and dairy products [4]. Thus, dietary boron intake varies widely depending on the proportions of fiber and protein-rich plant foods consumed and concentrations of boron in the soil and in drinking water [3–5]. A diverse, plant-food-rich diet is estimated to provide about 1.5–3.0 mg/day of boron, while reported values for the intake of dietary boron in the European Union range between 0.8 and 1.9 mg/day [4]. Boron is easily absorbed across the gastrointestinal tract and mucous membranes and primarily excreted in the urine [3].

Low as well as high boron status seems to be detrimental to human and animal health [1, 3, 7]. The exact required amount of boron in humans has not yet been determined [7]. Until now, neither an estimated average requirement nor a dietary reference intake value has been defined for boron. Only an upper intake level of 20 mg/day has been set for adults [4, 7]. However, boron is considered as a potentially essential mineral for human health. Biological roles of boron have not been fully explained yet and available evidence is primarily based on experimental studies, with human data being scarce [1, 3, 7]. Nevertheless, a wide range of physiological roles of boron have been suggested, with the trace mineral potentially (i) being, amongst others, essential for bone metabolism, (ii) enhancing absorption of other minerals (i.e. magnesium, calcium, phosphate), (iii) beneficially impacting the body’s use of estrogen, testosterone, and vitamin D, (iv) reducing concentrations of inflammatory biomarkers (e.g. C-reactive protein [CRP], tumor necrosis factor-α), (v) raising expression of genes encoding antioxidant enzymes, (vi) improving cognitive performance, (vii) demonstrating preventive and therapeutic effects in cancers, (viii) inhibiting adipogenesis, and (ix) modulating plasma insulin concentrations [1, 3, 4, 6–9].

In the light of these numerous potential health-related functions, but the lack of comprehensive human studies, we aimed to investigate the role of boron in human metabolism. In the present analyses, we measured plasma boron concentrations in a general population sample with the intention to (i) determine dietary patterns, which are associated with plasma boron concentrations and (ii) identify anthropometric and cardio-metabolic factors, which are related to plasma boron concentrations.

## Subjects and methods

### Study sample

**Fig. 1:**
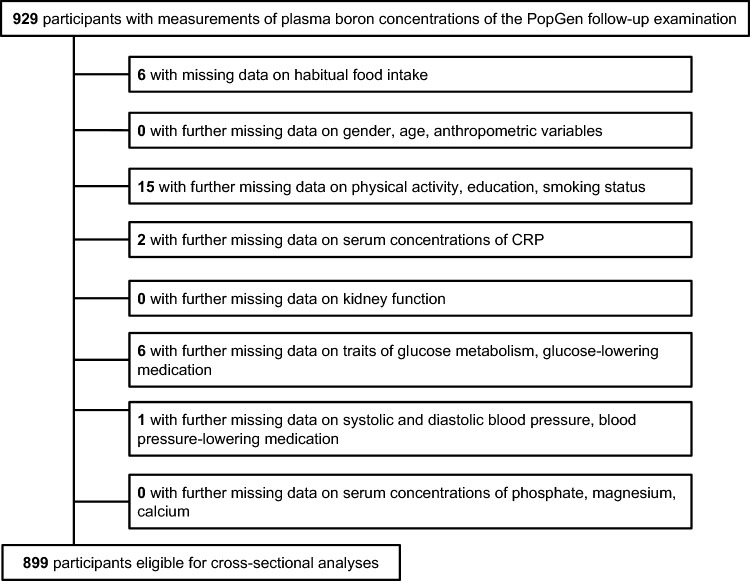
Flowchart showing the participants being eligible for analysis from those with measurements of plasma boron concentrations

The “PopGen controls” are a prospective population-based cohort, which was originally established as a reference (“control”) sample for genetic association analyses by the PopGen Biobank in Kiel, Germany. We recruited a total of 1317 residents of the city of Kiel and its surrounding communities in Northern Germany from a sample of individuals randomly selected from local population registries as well as from blood donors from the University Hospital in Kiel between 2005 and 2007. The age range of participants at the time of recruitment was between 19 and 77 years. As expected, participants randomly recruited from population registries were older and had higher prevalence of cardio-metabolic disease, but similar body mass index (BMI) as compared to individuals recruited from blood donors (*n* = 570) [10, 11]. The present cross-sectional analyses were based on data from the first follow-up examination, which was conducted between 2010 and 2012 and included physical examinations, laboratory analyses, and standardized questionnaires on demographics, lifestyle factors (including diet, physical activity, education, and smoking status), and medical history [12, 13]. For all of the 929 individuals participating in this follow-up examination, plasma boron concentrations were available. After exclusion of 30 individuals with missing data on e.g. dietary intake, physical activity, education, or smoking status, a total of 899 individuals were eligible for analyses on the associations of plasma boron concentrations with patterns of food intake and on the metabolic correlates of boron (Fig. 1).

The study was conducted in accordance with the Declaration of Helsinki. The study protocol was approved by the Ethics Committee of the Medical Faculty of Kiel University (Project identification code A 156/03; P2N reference numbers 2020-013, 2020-041). All participants provided written informed consent prior to their inclusion in the study.

### Clinical evaluation

Trained personnel performed anthropometric measurements in a standardized manner [14, 15]. The systolic and diastolic blood pressure values were calculated as the arithmetic mean of two blood pressure measurements, conducted using a sphygmomanometer with the participants sitting for at least 5 min and with at least 2 min between the measurements. Hypertension was defined as systolic blood pressure ≥ 140 mmHg or diastolic blood pressure ≥ 90 mmHg or use of antihypertensive medication [12, 14, 16].

Information about participant characteristics such as age, sex, years of education, smoking habits, and medical history were obtained using self-administered questionnaires [14, 15]. Participants additionally reported their habitual time (hours) spent in physical activity (walking, cycling, sports, gardening, housework, home repair, stair climbing) per week during the last 12 months [14]. From these questions, total physical activity was derived by summing up the metabolic equivalent of task (MET) values assigned to each corresponding activity [17].

### Laboratory analyses

Whole-blood and lithium heparin plasma samples were drawn for biochemical assessment from the participants in a sitting position. Concentrations of HbA1c, plasma glucose, CRP, plasma lipids (triglycerides, high-density lipoprotein [HDL] cholesterol, low-density lipoprotein [LDL] cholesterol), were analyzed on the same day in unfrozen blood samples with HbA1c being measured by high performance liquid chromatography (HPLC) with photometric detection (D-10 HPLC analyzer; Bio-Rad Laboratories, Munich, Germany), glucose using an enzymatic UV test (hexokinase method, Hitachi Modular; Roche Diagnostics, Mannheim, Germany), CRP by immunoturbidimetry, and lipids using enzymatic colorimetry (Hitachi Modular; Roche Diagnostics) [14, 15, 18]. Participants having CRP concentrations below the detection limit of 0.9 mg/L (*n* = 345) were assigned half of the detection limit as their CRP value. Prevalent diabetes was defined as HbA1c ≥ 6.5% (48 mmol/mol) or fasting plasma glucose ≥ 126 mg/dL or use of anti-diabetic medication [12]. Plasma creatinine concentrations were measured enzymatically on Roche/Hitachi cobas c systems (Roche Diagnostics). Estimated glomerular filtration rate (eGFR) was derived using the Chronic Kidney Disease Epidemiology Collaboration (CKD-EPI) equation [19].

Blood samples for biomarker analyses in plasma were drawn into plasma separator tubes (Sarstedt AG, Nümbrecht, Germany), centrifuged, aliquoted, and stored at − 80 °C until analyses [12]. Plasma boron, phosphate, magnesium, and calcium concentrations were measured via an inductively coupled plasma-mass spectrometry (ICPMS) ICAP Q instrument (Thermo Fisher Scientific, Waltham, MA, USA) at Synalb (Jena, Germany). Measurements were conducted in accordance with DIN EN ISO 17294-2: 2017-01. Samples were decomposed with a mixture of nitric acid and hydrogen peroxide (4:1) using microwave pressure digestion. For calibration, a multi-element standard was used. To minimize spectroscopic interferences, a collision/reaction cell was used for detection. Rhodium (2 µg/L) was added as the internal standard.

### Assessment of dietary intake

Dietary assessment within the PopGen cohort has been described in detail before [12–15, 18]. In brief, habitual dietary intake over the past 12 months was assessed using a self-administered 112-item food frequency questionnaire, which has been validated within the European Prospective Investigation into Cancer and Nutrition (EPIC)-Potsdam study [20]. On the basis of the intake frequencies and quantities of foods consumed, food group intake, nutrient content, and energy intake were calculated using the German Food Code and Nutrient Database (version II.3) [20, 21].

### Statistical analyses

SAS (version 9.4; SAS Institute, Cary, NC) procedures were used for data analyses and *P* values < 0.05 were considered statistically significant.

Categorical variables were reported as absolute numbers and percentages, normally distributed continuous variables as mean and standard deviation (SD), and continuous skewed variables as median and interquartile range (IQR). Study participants’ characteristics were compared across tertiles of plasma boron concentration using chi-square test for categorical variables, general linear models for continuous normally distributed variables, and Kruskal–Wallis test for continuous skewed variables (Table 1).

**Table 1:** General characteristics of the overall study sample and according to tertiles of plasma boron concentrations (*n* = 899)

	Total	*T*1 ≤ 28.26 µg/L	*T*2 >28.26–40.28 µg/L	*T*3 ≥ 40.29 µg/L	P*
*n* (% male)^a^	899 (57)	299 (62)	300 (55)	300 (54)	0.106
Plasma concentration of boron (µg/L)^b^	33.80 (25.61; 44.65)	**22.76 (19.45; 25.61)**	**33.79 (30.84; 37.08)**	**51.06 (44.64; 61.30)**	**<0.001**
Age (years)^c^	61 ± 13	**54 ± 13**	**62 ± 11**	**65 ± 11**	**<0.001**
Body mass index (kg/m^2^)^c^	27.4 ± 4.7	**28.4 ± 5.5**	**27.2 ± 4.2**	**26.6 ± 4.2**	**<0.001**
Waist circumference (cm)^c^					
Male	100.9 ± 11.3	101.9 ± 12.3	100.6 ± 10.5	100.0 ± 10.7	0.285
Female	90.9 ± 13.8	**93.3 ± 15.5**	**91.2 ± 13.3**	**88.6 ± 12.5**	**0.026**
Waist-to-hip ratio^c^					
Male	0.99 ± 0.07	0.99 ± 0.07	0.99 ± 0.06	0.98 ± 0.06	0.428
Female	0.88 ± 0.08	0.88 ± 0.08	0.87 ± 0.07	0.88 ± 0.08	0.835
C-reactive protein (mg/L)^b^	1.20 (0.45; 2.60)	1.40 (0.45; 3.10)	1.20 (0.45; 2.70)	1.15 (0.45; 2.10)	0.137
Estimated glomerular filtration rate (mL/min/1.73 m^2^)^c^	84.5 ± 15.2	**91.4 ± 13.5**	**83.6 ± 14.0**	**78.5 ± 15.3**	**<0.001**
Plasma creatinine concentration (mg/dL)^c^	0.88 ± 0.18	**0.86 ± 0.16**	**0.88 ± 0.17**	**0.92 ± 0.21**	**<0.001**
HbA1c (%)^c^	5.7 ± 0.6	5.7 ± 0.7	5.7 ± 0.6	5.8 ± 0.6	0.272
Plasma glucose concentration (mg/dL)^b^	98.0 (91.0; 105.0)	**95.0 (89.0; 104.0)**	**98.5 (91.5; 105.0)**	**99.0 (93.0; 105.0)**	**<0.001**
Prevalent diabetes^a^	81 (9.0)	28 (9.4)	21 (7.0)	32 (10.7)	0.282
Glucose-lowering medication (yes/no)^a^	38 (4.2)/861 (95.8)	11 (3.7)/288 (96.3)	12 (4.0)/288 (96.0)	15 (5.0)/285 (95.0)	0.704
Plasma triglyceride concentration (mg/dL)^b^	105.0 (77.0; 140.0)	107.0 (78.0; 144.0)	103.0 (75.5; 136.5)	104.5 (76.5; 140.0)	0.364
Plasma high-density lipoprotein cholesterol concentration (mg/dL)^c^	65.0 ± 18.1	**60.9 ± 15.9**	**65.6 ± 17.9**	**68.6 ± 19.6**	**<0.001**
Plasma low-density lipoprotein cholesterol concentration (mg/dL)^c^	131.3 ± 33.5	129.0 ± 32.3	134.3 ± 34.2	130.7 ± 33.7	0.141
Lipid-lowering medication (yes/no)^a^	118 (13.1)/781 (86.9)	28 (9.4)/271 (90.6)	44 (14.7)/256 (85.3)	46 (15.3)/254 (84.7)	0.060
Systolic blood pressure (mmHg)^c^	84.8 ± 9.0	139.4 ± 17.3	139.2 ± 18.6	140.8 ± 19.2	0.518
Diastolic blood pressure (mmHg)^c^	139.8 ± 18.4	85.0 ± 9.1	84.5 ± 8.9	84.9 ± 8.9	0.821
Antihypertensive medication (yes/no)^a^	222 (24.7)/677 (75.3)	**46 (15.4)/253 (84.6)**	**78 (26.0)/222 (74.0)**	**98 (32.7)/202 (67.3)**	**<0.001**
Prevalent hypertension^a^	563 (62.6)	182 (60.9)	181 (60.3)	200 (66.7)	0.206
Current smokers^a^	125 (13.9)	**57 (19.1)**	**42 (14.0)**	**26 (8.7)**	**0.002**
Alcohol intake (g/day)^b^	8.9 (3.2; 18.4)	**6.8 (2.6; 16.0)**	**8.1 (3.2; 15.7)**	**12.3 (4.5; 21.9)**	**<0.001**
Physical activity (MET-hours/week)^b^	89.0 (57.6; 130.5)	**80.1 (50.0; 121.3)**	**92.0 (59.6; 130.5)**	**93.6 (60.4; 139.3)**	**0.012**
Education level (low [< 10 years], medium [10 years], high [≥ 11 years])^a^	314 (34.9)/293 (32.6)/292 (32.5)	113 (37.8)/98 (32.8)/88 (29.4)	112 (37.3)/93 (31.0)/95 (31.7)	102 (34.0)/89 (29.7)/109 (36.3)	0.179
Plasma phosphate concentration (mg/L)^c^	141.0 ± 20.3	**136.3 ± 19.5**	**141.6 ± 20.1**	**145.2 ± 20.4**	**<0.001**
Plasma magnesium concentration (mg/L)^c^	20.5 ± 1.7	20.4 ± 1.7	20.6 ± 1.6	20.6 ± 1.8	0.208
Plasma calcium concentration (mg/L)^c^	97.9 ± 7.2	**97.1 ± 7.0**	**98.0 ± 7.2**	**98.7 ± 7.4**	**0.023**

Bold indicates significant associations (*P* < 0.05)

Values are ^a^*n* (%), ^b^median (*Q*1; *Q*3), ^c^mean ± SD

*CI* confidence interval, *MET* metabolic equivalent of task, *T* tertile

*P values based on chi-square test (categorical variables), Kruskall–Wallis test (continuous skewed variables) or general linear models (continuous normally distributed variables)

For the assessment of seasonal variations in plasma boron concentrations, categories corresponding the four seasons and based on the month of study examination (winter, January–March; spring, April–June; summer, July–September; fall, October–December) were created [12]. Plasma boron concentrations were presented as median and IQR with the *P* value for comparison across the seven seasons calculated using Kruskall–Wallis test (Table 2).

**Table 2:** Seasonal differences in plasma boron concentrations (*n* = 899)

	Spring (*n* = 167)	Summer (*n* = 168)	Autumn (*n* = 224)	Winter (*n* = 340)	P*
Plasma concentration of boron (µg/L)	33.8 (26.0; 43.3)	36.9 (28.7; 48.3)	35.4 (26.4; 47.2)	31.4 (23.4; 42.3)	0.002

Values are median (*Q*1; *Q*3).

*P values based on Kruskall Wallis test

For presentation of dietary intake according to tertiles of plasma boron concentrations, the 112 food items of the FFQ were assigned to 42 predefined food group categories according to similarity of nutrient characteristics and/or culinary usage for simplification of data interpretation and minimization of within-person variations in individual food intakes [12, 14] in a first step. Food group intakes were then ln-transformed prior to analysis due to their skewed distribution and presented as re-transformed least square means and 95% confidence interval (CI) adjusted for age and sex. To allow ln-transformation of food groups including non-consumers (i.e. beer, poultry, beef), a positive constant of 0.1 was added to the respective food groups. Age- and sex-adjusted differences in food group intakes by tertiles of plasma boron concentrations were calculated using general linear models (Supplemental Table 1).

#### Calculation of plant-based diet indices

Plant-based diet indices were derived according to Satija et al. [22, 23] and as previously reported for the PopGen cohort by Ratjen et al. [13]. Briefly, the 112 items of the FFQ were assigned to 18 food groups representing the following 3 food categories: (1) healthy plant foods (i.e. whole grains, fruits, vegetables, nuts, legumes, vegetable oils, tea/coffee), (2) less healthy plant foods (i.e. fruit juices, refined grains, potatoes, sugar-sweetened beverages, sweets/desserts), and (3) animal foods (i.e. animal fat, dairy, eggs, fish/seafood, meat, miscellaneous animal-based foods) (for more details on categorization of the FFQ-items into the food groups see [13]). Of note, plant foods were defined as healthy or less healthy based on existing knowledge on their association with chronic disease outcomes; those plant foods which were not clearly assignable to one of these two groups (e.g. alcoholic beverages) were not included in the indices [13, 22]. Instead, multivariable regression analyses were adjusted for alcohol intake [13]. Sex-specific positive (*Q1* = 1 and *Q5* = 5) and reverse (*Q1* = 5 and *Q5* = 1) quintile scores were assigned to each of the 18 food groups. The overall plant-based diet index (PDI) was then generated by summing up positive scores of the healthy and less healthy plant food categories and reverse scores of the animal food category. For the healthy plant-based diet index (hPDI), positive scores of the healthy plant foods and reverse scores of the less healthy plant and animal foods were summed up. For scoring of the unhealthy plant-based diet index (uPDI), positive scores for the less healthy plant food category and reverse scores for the healthy plant and animal food categories were summed up. Thus, the three plant-based diet indices may range from 18 to 90 with a higher index reflecting a more plant-based (either overall, healthy or unhealthy) and less animal-based diet [13, 22, 23].

#### Dietary pattern derivation by reduced rank regression

Additionally to the application of the hypothesis-based approach of deriving dietary patterns, i.e. the plant-based diet indices, reduced rank regression (RRR) was used to identify a dietary pattern explaining maximal variation in circulating boron concentrations. RRR applied in nutritional epidemiology is a statistical method, which determines linear functions of predictors (i.e. foods) by maximizing the explained variation in predefined response variables (i.e. disease-related intermediate variables) [24]. In the present analysis, plasma boron concentrations were chosen as response variable. As the number of dietary patterns extracted by RRR equals the number of response variables chosen [24], we derived one dietary pattern. RRR analysis was performed using the RRR option in the SAS procedure PLS [24] using ln-transformed and sex-standardized values of the 42 predefined food group categories as predictor variables and ln-transformed plasma boron concentrations as response variable. As described above, a positive constant of 0.1 was added to the food groups including non-consumers (i.e. beer, poultry, beef). Food groups with absolute factor loadings (i.e. correlations between the extracted factor and foods) ≥ 0.15 were considered as contributing to the dietary pattern [12]. Finally, the RRR factor score was calculated according to the simplified pattern approach to reduce population dependency of the dietary pattern [25].

#### Regression models with dietary indices/pattern

Associations between plasma boron concentrations as continuous dependent variable and dietary indices, modelled per ten-point increment in PDI, hPDI, and uPDI, respectively, were assessed using multivariable-adjusted linear regression analyses, yielding regression coefficients with their corresponding 95% CI as well as *P* values. In addition, adjusted means of circulating boron concentrations were calculated according to tertiles of the dietary indices (with a higher tertile indicating higher adherence to the dietary indices) to obtain intuitive values for presentation of effect sizes [26]. Plasma boron concentrations were ln-transformed prior to analysis and back transformed for presentation of the data. Model 1 was adjusted for age (years) and sex. Model 2 was additionally adjusted for physical activity (MET-h/wk), smoking status (never, former, current), education level (≤ 9, 10, ≥ 11 years), season (winter, spring, summer, fall), total daily energy intake (kJ/day), total daily alcohol intake (g ethanol/day); with energy and alcohol intake being ln-transformed prior to analysis. For model 3, BMI was added as additional confounder.

By definition, as the RRR pattern has been derived for plasma boron concentrations as response variable, it will be predictive for this variable. However, to (i) illustrate the effect sizes of the association and (ii) investigate whether the obtained RRR pattern is still predictive after adjustment for potential confounders [27], multivariable-adjusted linear regression analysis as described for the plant-based diet indices, modelling a 1-unit increment in the RRR pattern, was conducted.

#### Interaction analysis

Differences in the slopes of hPDI and uPDI in their association with plasma boron concentrations were tested using linear regression analyses with the estimate statement of proc glm.

#### Anthropometric and cardio-metabolic correlates of circulating boron

To identify non-dietary correlates of plasma boron concentrations, we performed a stepwise forward selection linear regression model with a *P* ≤ 0.1 for model entry, using ln-transformed boron concentrations as the dependent variable. Potential correlates were age, sex (both forced into the model), BMI, waist-to-hip ratio (WHR), CRP concentrations (ln-transformed), eGFR, HbA1c, systolic blood pressure, plasma concentrations of triglycerides (ln-transformed), HDL-cholesterol, LDL-cholesterol, total phosphate, magnesium, and calcium and possible confounders, i.e. physical activity (MET-h/wk), smoking status (never, former, current), education level (≤ 9, 10, ≥ 11 years), season (winter, spring, summer, fall), and glucose-lowering, antihypertensive, and lipid-lowering medication (all yes/no). We present a final multivariable-adjusted model, using ln-boron as the outcome (dependent) variable, with age, sex, and all those variables in the model, that were significant in the forward selection procedure.

#### Restricted cubic splines regression

To assess nonlinear associations of dietary indices/pattern or anthropometric/cardio-metabolic variables with plasma boron concentrations, restricted cubic splines regression (RCS) was applied using the adjustment set of the respective fully adjusted model as described above (dietary indices/pattern) as well as all variables of the forward selection regression model, respectively.

#### Power calculations

Post hoc power calculations and participant numbers needed to detect changes (Supplemental Table 2) were conducted as described in Supplemental Material 1.

## Results

### Participant characteristics

Of the 899 participants eligible for analyses (Fig. 1), 57% were men and the mean age of this elderly sample was 61 years (Table 1). Overall, the average mean BMI of the participants was in the overweight range and hypertension was present in almost two-thirds of the study sample. The median plasma boron concentration of the total study sample was within the range of previously reported mean plasma boron concentrations of about 17–80 µg/L [6]. Compared to the bottom tertile, individuals in higher tertiles of plasma boron concentrations were older, had a higher daily alcohol intake, higher plasma glucose concentrations, and a lower eFGR, with only individuals within the lowest tertile of plasma boron concentrations having on average a normal kidney function [28]. However, individuals in the highest compared to lower tertiles of plasma boron concentrations had a lower BMI, a higher level of physical activity, and were less likely current smokers (Table 1). Circulating boron varied according to the four seasons and were highest during summer and lowest during winter (Table 2).

### Nutritional correlates of circulating boron

Individuals in the highest compared to the lowest tertile of plasma boron concentrations had higher age- and sex-adjusted daily mean intakes of the following plant-based food groups: leafy and root vegetables, fruits, nuts and seeds, cereals other than bread/pasta/rice, vegetable oils, and soya products and lower intakes of the following animal-based food groups: pork, poultry, processed meat, and other fats. Regarding mean beverage intake, individuals in the highest compared to the lowest tertile of plasma boron concentrations had lower intakes of soft drinks and higher intakes of non-alcoholic beverages, tea, and wine (Supplemental Table 1).

Additionally, RRR analysis identified a dietary pattern explaining about 30% of the variation in plasma boron concentrations. This RRR-derived dietary pattern was characterized by high intakes of fruits, nuts and seeds, tea and wine and low intakes of bread, poultry, processed meat, margarine, chocolate and sweets, soft drinks, sauces, and snacks (Table 3). As expected, the RRR pattern was strongly and independently associated with plasma boron concentrations: a 1-unit increase in the RRR pattern was associated with a 2.7% (95% CI: 2.1; 3.3), *P* < 0.001 (model 3) increase in circulating plasma boron concentrations.

**Table 3:** Factor loadings in 42 food groups and explained variation in ln-transformed boron plasma concentration by using reduced rank regression (*n* = 899)

	Reduced rank regression factor loading
	Overall
Explained variance in food intake (%)	5.0
Explained variance in response variable (plasma boron concentration) (%)	29.5
Food group (g/day)	
Potatoes	–
Leafy vegetables	–
Fruiting vegetables	–
Root vegetables	–
Cabbage	–
Other vegetables	–
Legumes	–
Fruits	0.2484
Nuts and seeds	0.2591
Milk	–
Dairy products	–
Cheese	–
Bread	−0.1973
Pasta and rice	**–**
Other cereals (flour, flakes, starches, semolina, dough and pastry, breakfast cereals)	**–**
Beef	**–**
Pork	**–**
Poultry	−0.2339
Processed meat	−0.2122
Other meat (offals, hash, other meat)	**–**
Fish, fish products	**–**
Eggs	**–**
Butter	**–**
Margarine	−0.2146
Vegetable oils	**–**
Other fats	**–**
Sugar products (e.g. syrups, candy, ice cream, desserts)	**–**
Chocolate, sweets	−0.1725
Cake, cookies	**–**
Non-alcoholic beverages	**–**
Soft drinks	−0.4040
Coffee	
Tea	0.1670
Beer	**–**
Wine	0.3805
Other alcoholic beverages	**–**
Sauces	−0.1516
Soups	**–**
Bouillon	–
Soya products	–
Dietetic products	–
Snacks	−0.2458

Food groups with absolute factor loadings ≥ 0.15 were considered as contributing to the dietary pattern. Ln-transformed and standardized food groups were used as predictor variables and ln-transformed plasma boron concentrations as response variable for reduced rank regression

Increases in PDI and hPDI were associated with increases in circulating boron concentrations, while an increase in uPDI was related to a reduction in circulating boron (Table 4). RCS analysis did not reveal any statistically significant nonlinear associations between plant-based diet indices and plasma boron concentrations (all *P* > 0.05). The test for differences in slopes revealed differences between hPDI and uPDI in their association with plasma boron concentrations (*P* = 0.002).

**Table 4:** Associations of plant-based diet index with plasma boron concentrations (n=899)

	Least square mean and 95% CI in plasma boron concentrations by tertiles of plant-based diet indices			Percentage change (estimate and 95% CI) in plasma boron concentrations per 10-point increase in plant-based diet indices	
	T1	T2	T3	*β* (95% CI)^a^	P*
Plant-based diet index					
Model 1	32.9 (31.5; 34.4)	33.9 (32.3; 35.5)	36.6 (35.1; 38.2)	8.7 (4.2; 13.3)	<0.001
Model 2	33.0 (31.5; 34.5)	33.9 (32.4; 35.5)	37.3 (35.6; 39.0)	9.6 (5.0; 14.4)	<0.001
Model 3	32.9 (31.5; 34.5)	34.0 (32.5; 35.6)	36.8 (35.2; 38.5)	8.7 (4.2; 13.4)	<0.001
Healthy plant-based diet index					
Model 1	31.9 (30.5; 33.4)	34.2 (32.8; 35.7)	37.6 (36.0; 39.4)	11.5 (7.8; 15.4)	<0.001
Model 2	32.2 (30.7; 33.7)	34.8 (33.3; 36.3)	37.7 (36.0; 39.6)	11.2 (7.4; 15.2)	<0.001
Model 3	32.2 (30.8; 33.7)	34.7 (33.2; 36.2)	37.4 (35.7; 39.2)	10.4 (6.6; 14.3)	<0.001
Unhealthy plant-based diet index					
Model 1	36.6 (35.0; 38.3)	35.4 (33.9; 36.9)	31.7 (30.4; 33.1)	−8.5 (−11.7; −5.2)	<0.001
Model 2	36.9 (35.2; 38.7)	35.4 (33.9; 37.0)	32.1 (30.6; 33.6)	−8.7 (−12.1; −5.2)	<0.001
Model 3	36.8 (35.1; 38.5)	35.4 (33.8; 36.9)	31.9 (30.4; 33.4)	−8.8 (−12.1; −5.4)	<0.001

Data are least square means with 95% CIs of plasma boron concentrations by tertiles of dietary indices and regression coefficients (*β*) with 95% CIs and *P values from linear regression analyses with dietary indices as the independent variable

*CI* confidence interval, *T* tertile

Model 1 adjusted for age, sex. Model 2 additionally adjusted for physical activity, smoking status, education level, season, total daily energy and alcohol intake (both ln-transformed). Model 3 additionally adjusted for BMI

Boron entered into the models as ln-transformed variable

^a^Regression coefficients should be interpreted as follows: A ten-point increase in plant-based diet indices associates with a %-change in plasma boron concentrations by *β*

### Association of plasma boron concentrations with cardio-metabolic traits

**Fig. 2:**
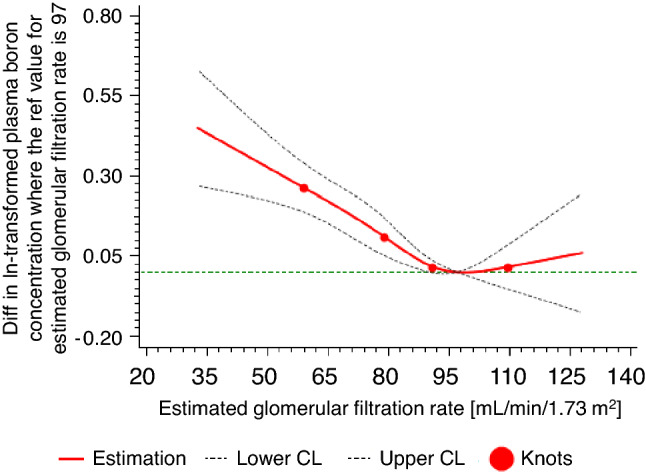
**Nonlinear associations of plasma boron concentrations with estimated glomerular filtration rate using restricted cubic splines** (***n*** **= 899**). Restricted cubic splines (RCS) regression for the association of ln-transformed plasma boron concentrations with estimated glomerular filtration rate, representing the association presented in Table 5 with a significant nonlinear association. The solid line indicates estimated differences in the respective dependent variable and dashed lines indicate 95% CI from RCS regression, with four knots placed at the 5th, 35th, 65th and 95th percentiles of the distribution, using 97 mL/min/1.73 m^2^ as a reference of estimated glomerular filtration rate. Estimated differences were adjusted for all other covariates identified using stepwise forward selection procedures as indicated in Table 5. Wald *P* values are *P* for nonlinearity = 0.014 and *P* for overall association < 0.001

In a forward selection process, the variables displayed in Table 5 were identified as statistically significant correlates of plasma boron concentrations explaining 31% of the inter-individual variation in plasma boron concentrations. BMI, CRP, and plasma lipid concentrations displayed inverse associations, whereas age and plasma phosphate concentrations were positively associated with boron levels. Circulating boron concentrations displayed a nonlinear, U-shaped association with eGFR (Table 5). Below a threshold of about 97 mL/min/1.73 m^2^, eGFR was inversely associated with plasma boron concentrations, while above this threshold, higher eGFR was associated with higher plasma boron concentrations (Fig. 2). Also, plasma boron concentrations were higher in individuals with high education level and medium education level vs. low education level, in summer vs. winter, and in individuals taking antihypertensive medication vs. those taking no antihypertensive medication. Anthropometric and cardio-metabolic traits which were not identified as significant correlates of plasma boron concentrations were gender, WHR, HbA1c, systolic blood pressure, plasma magnesium and calcium concentrations, physical activity level, smoking status, and glucose- and lipid-lowering medication.

**Table 5:** Anthropometric and cardio-metabolic correlates of plasma boron concentrations as determined by linear regression analysis (n=899)

	Percentage change (estimate and 95% CI) in plasma boron concentrations			
	*β* (95% CI)	P*	*P*_overall_^*ǂ*^	*P*_nonlinear_^*ǂ*^
Age (per 5 year increase)^a^	4.79 (3.43; 6.16)	–	**<0.001**	0.845
Men (vs. women)^b^	−2.29 (−7.47; 3.18)	0.404	–	–
Body mass index (per 3 kg/m^2^ increase)^a^	−2.84 (−4.53; −1.12)	–	**0.002**	0.101
C-reactive protein (per 50% increase)^b^	−1.13 (−2.09; −0.17)	–	**0.025**	0.140
Estimated glomerular filtration rate (per 10 ml/min/1.73 m^2^ increase)^a^	−5.87 (−7.68; −4.02)	–	**<0.001**	**0.014**
Plasma triglyceride concentration (per 10% increase)^b^	−1.50 (−2.18; −0.81)	–	**<0.001**	0.067
Plasma high-density lipoprotein cholesterol concentration (per 5 mg/dL increase)^a^	−1.28 (−2.42; −0.14)	–	**0.029**	0.127
Plasma low-density lipoprotein cholesterol concentration (per 10 mg/dL increase)^a^	−2.12 (−2.93; −1.29)	–	**<0.001**	0.629
Plasma total phosphate concentration (per 15 mg/L increase)^a^	13.24 (9.88; 16.71)	–	**<0.001**	0.711
High education level (vs. low education level)^b^	18.42 (11.67; 25.58)	**<0.001**	–	–
Medium education level (vs. low education level)^b^	9.32 (3.3; 15.68)	**0.002**	–	–
Spring (vs. winter)^b^	1.56 (−4.9; 8.46)	0.644	–	–
Summer (vs. winter)^b^	9.98 (2.96; 17.49)	**0.005**	–	–
Fall (vs. winter)^b^	1.26 (−4.71; 7.6)	0.686	–	–
Taking antihypertensive medication (vs. taking no antihypertensive medication)^b^	9.54 (3.03; 16.46)	**0.004**	–	–

Data are regression coefficients (*β*), 95% CIs, *P values from linear regression analyses ^*ǂ*^and restricted cubic splines regression with plasma boron concentrations as dependent variable

The multivariable model explained 31% of the inter-individual variation in plasma boron concentrations

*CI* confidence interval

^a^Independent variables not transformed before analysis, ^b^binary variables; boron and ^c^independent variables entered into the models as ln-transformed variables. Boldface indicates significant associations (*P* < 0.05)

Regression coefficients should be interpreted as follows: ^a^An *x*-unit increase in the independent variable associates with a relative (%) change of plasma boron concentrations by *β*. ^b^Relative (%) increase or decrease in plasma boron concentrations by *β* for presence vs. absence of the respective trait. ^c^A relative (10% or 50%-fold) increase in the independent variable associates with a relative (%) change of plasma boron concentrations by *β*

## Discussion

In a moderate-sized community-based sample, we assessed nutritional and cardio-metabolic correlates of plasma boron concentrations. Our main observations were as follows. First, we identified a dietary pattern explaining about 30% of the inter-individual variation in circulating boron. This RRR pattern was characterized by high intakes of plant foods presumed to be healthy and low intakes of less healthy plant foods and animal foods. Second, plasma boron concentrations were inversely related to BMI, CRP, and plasma lipid concentrations and directly related to age and plasma phosphate concentrations. eGFR showed a U-shaped association with circulating boron. In total, the identified traits explained 31% of the inter-individual variation in circulating boron. Third, plasma boron concentrations showed seasonal variations.

## In the context of the published literature

### Nutritional correlates of plasma boron concentrations

Food group intake across tertiles of circulating boron within this study largely mirrored the known categorization of foods into good (i.e. fibre- and protein-rich plant foods, wine) and poor (i.e. animal foods) sources of boron. Only the higher intakes of dairy products, cheese, and fish in the highest compared to the lowest tertile of plasma boron concentrations contrasts prior evidence [3, 4, 6], but appear plausible in the light of the plant-derived boron intake of cows, which is transferred into the cows’ milk dose-dependently with the cows’ boron ingestion [29, 30]. Higher plasma boron concentrations in our study were not only associated with a closer adherence to the PDI and hPDI, but additionally with a more favorable lifestyle in general as participants in a higher compared to a lower tertile of circulating boron were less likely to be current smokers and more likely to be physically active. Also, individuals with high or medium vs. low education level showed higher circulating boron, which might be also mediated by healthier food choices.

### Cardio-metabolic correlates of plasma boron concentrations

We identified a multivariable-adjusted model that explained 31% of the inter-individual variation in plasma boron concentrations. This model included age, BMI, CRP, eGFR, plasma lipid and phosphate concentrations, education level, season, and antihypertensive medication.

Our observation of an inverse association of BMI with circulating boron is comparable with results from experimental studies showing a reduction in body weight and obesity with boron administration in animal models [31, 32]. Proposed mechanisms are the overexpression of thermogenic proteins in murine adipose and skeletal muscle tissue, resulting in accelerated lipolysis and body weight loss [33]. Studies in cell models suggested that boron might suppress adipogenesis [34, 35]. In agreement with this experimental evidence and with our observations, a small intervention study in 13 healthy women reported that an increase in dietary boron intake for one month by means of boron-rich foods decreased body weight and BMI [36].

Boron has been also suggested to reduce systemic inflammation, as indicated by associations of lower concentrations of inflammatory biomarkers such as CRP with higher boron concentrations in our sample and as reviewed elsewhere [4, 7]. Similar observations have been reported in a further human study, where CRP concentrations decreased after supplementation with calcium fructoborate for 30 days in 78 healthy individuals [37]. The dosages of boron used in these intervention studies ranged from 3.0 to 12.0 mg boron/day [4, 7, 37], which is above the 1.5–3.0 mg/day of dietary boron intake reported to be achievable by consuming a diverse, plant-food-rich diet, but clearly below the tolerable upper intake level of 20 mg boron/day for adults [4, 7].

We observed a relatively pronounced inverse association of eGFR with plasma boron concentrations for individuals with eGFR <97 mL/min/1.73 m^2^. The most likely explanation is that boron is mainly excreted through the kidney [3]. Indeed, the above-mentioned human study by Kuru et al. reported a 6.0-fold increase in urinary boron concentrations with rising dietary boron intake [36]. As comprehensively summarized by Pahl et al., chronic exposure to nonlethal doses of boric acid in humans does not seem to be associated with renal abnormalities [38]. Therefore, the present results might rather reflect a reduced renal clearance, thus higher concentrations of boron within the body in individuals with slightly reduced kidney function, than pointing towards a direct detrimental effect of boron on renal function.

In animal studies, boron supplementation reduced circulating concentrations of triglycerides and cholesterol and, thus, improved plasma lipid profiles [3, 7]. These positive effects of boron appear to be also present in humans as we observed inverse associations of circulating boron with circulating triglycerides and LDL-cholesterol concentrations. However, circulating boron was additionally inversely related to HDL-cholesterol concentrations in our sample.

So far, the cardio-protective benefits of (red) wine consumption have been commonly attributed to its polyphenol and/or alcohol content [39]. However, taking together our associative findings of higher intakes of wine in the highest (least square mean [95% CI]: 55.1 g/day [47.3; 64.2]) compared to the lowest (least square mean [95% CI]: 20.8 g/day [17.1; 24.3]) tertile of plasma boron concentrations (see also Supplemental Table 1) and the cardio-metabolic correlates of plasma boron concentrations, these cardio-protective benefits might be (at least partially) mediated by boron.

### Seasonal variation of plasma boron concentrations

Our observed seasonal variations of plasma boron concentrations might reflect its previously described interactions with vitamin D metabolism [1, 4]. It has been hypothesized that boron supplementation increases the biological half-live and bioavailability of vitamin D [4]. A bi-directional regulation of boron and vitamin D or a lower intake of plant-based, thus boron-rich foods, during winter compared to summer might possibly explain our observations. Also, experimental and human evidence suggested that boron supplementation improves absorption and reduces excretion of phosphate, calcium, and magnesium [1, 3, 4], which is reflected by direct associations of circulating phosphate with circulating boron in our study. Of note, the hypothesis of boron influencing renal phosphate excretion is additionally supported by a partial tubular reabsorption of boron as observed in humans and animals [38]. Through boron’s interplay with phosphate and vitamin D, it is likely to exert essential functions on bone metabolism and prevention of osteoporosis [1, 3, 4, 6, 7].

### Strengths and limitations

Strengths of our study include the moderate-sized population-based sample and the comprehensive assessment of cardio-metabolic traits. Furthermore, we assessed dietary intake using a validated FFQ and derived both, an explorative dietary pattern explaining about 30% of the variation in plasma boron concentrations and three predefined plant-based diet indices using continuous scores. Also, we applied RCS regression to model non-/linear relationships. The following limitations merit consideration. First, boron has several postulated biological functions [1, 3, 4, 6–8], of which we were able to investigate many (e.g. interaction with minerals, biomarkers of systemic inflammation, and kidney function), but we had no sufficient data on e.g. arthritis, osteoporosis, and activity of antioxidant enzymes available. Second, dietary boron intake could not be directly calculated, as robust databases for boron amounts in foods are lacking [3]. We thus used the diet indices/pattern as surrogate parameters of dietary boron intake. However, plasma boron concentrations and FFQ-derived proxies for boron intake refer to different time intervals (i.e. the FFQ covered food intake during the last year [20], half-life of circulating boron after dietary intake is about 21 h [7]). Third, a boron-rich diet seems to be characterized by high intakes of foods presumed to be healthy [36]. Although organic boron-containing compounds might itself contribute to a reduced risk of cardiovascular diseases [40, 41], residual confounding by other ingredients cannot be ruled out [36]. Importantly, boron is also contained in pesticides and inorganic fertilizers [42] and might be ingested from these sources via conventionally grown fruits and vegetables. In our study, we cannot distinguish between organically versus conventionally grown fruits and vegetables to investigate the impact of pesticide-derived boron intake. Thus, further studies are needed regarding the influence of the farming method on the boron content in fruits and vegetables.

## Conclusion

In our elderly population-based sample, a boron-rich diet appeared to be characterized by high intakes of plant foods presumed to be healthy, low intakes of plant foods presumed to be less healthy, and low intakes of all kinds of animal foods. Higher plasma boron concentrations were related to lower BMI and circulating concentrations of CRP. Furthermore, plasma boron concentrations were associated with age, phosphate, and plasma lipid metabolism and showed seasonal variations. Human intervention studies are warranted to derive causal relationships of circulating and dietary boron with human health and metabolism. To facilitate the investigation of dietary boron intake in human studies, robust databases on the boron content of foods are needed and clarification of the non-/essentiality of the trace element boron for human health will form the basis to derive recommendations for a dietary boron intake being sufficient to exert boron’s proposed beneficial physiological roles.

## Supplementary Information

Below is the link to the electronic supplementary material.

Supplementary file1 (DOCX 26 KB)

## Abbreviations

BMI: Body mass index
CI: Confidence interval
CKD-EPI: Chronic Kidney Disease Epidemiology Collaboration
CRP: C-reactive protein
eGFR: Estimated glomerular filtration rate
EPIC: European Prospective Investigation into Cancer and Nutrition
hPDI: Healthy plant-based diet index
HPLC: High performance liquid chromatography
ICPMS: Inductively coupled plasma-mass spectrometry
IQR: Interquartile range
MET: Metabolic equivalent of task
PDI: Overall plant-based diet index
RCS: Restricted cubic splines
RRR: Reduced rank regression
uPDI: Unhealthy plant-based diet index

## Declarations

### Data availability

Data described in the article, code book, and analytic code will be made available upon reasonable request.
