# Supplementary material for: Plasma boron concentrations in the general population: a cross-sectional analysis of cardio-metabolic and dietary correlates
Source: Eur J Nutr. 2021 Nov 26;61(3):1363–75. doi: 10.1007/s00394-021-02730-w (PMC8921125; doi:10.1007/s00394-021-02730-w)
Supplement: Supplementary file 1 — Supplementary file1 (DOCX 26 KB) [file 394_2021_2730_MOESM1_ESM.docx]

**SUPPLEMENTAL MATERIALS**

**Title:** Plasma boron concentrations in the general population: A cross-sectional analysis of cardio-metabolic and dietary correlates

**Journal name:** European Journal of Nutrition

**Author names:** Katharina S. Weber, Ilka Ratjen, Janna Enderle, Ulrike Seidel, Gerald Rimbach, Wolfgang Lieb

**Corresponding author:** Katharina S. Weber, Institute of Epidemiology, Kiel University, Niemannsweg 11, 24105 Kiel, Germany; Email: [katharina.weber@epi.uni-kiel.de](mailto:katharina.weber@epi.uni-kiel.de)

**Supplemental Material 1.** Statistical methods used to conduct power and sample size calculations.

Post-hoc power calculations and participant numbers needed to detect changes were conducted considering the observed partial correlations. Participant numbers to detect significant differences were calculated for α=0.05 and a power of 80% and based on the observed partial correlations. Additionally, the actual power of the partial correlations as observed in our sample for a sample size of n=899 participants was determined (Supplemental Table 2).

**Supplemental Table 1**. Age- and sex-adjusted mean intake of 42 food groups [g/day] and adherence to the plant-based diet indices according to tertiles of plasma boron concentrations (n=899)

|  | Plasma concentration of boron [µg/L] | | |  |
| --- | --- | --- | --- | --- |
| Food groups [g/day] | T1  ≤ 28.26 µg/L  n=299 | T2  ≥28.26 – 40.28 µg/L  n=300 | T3  ≥ 40.29 µg/L  n=300 | *P_trend_** |
| Potatoes | 68.8 (64.0; 73.9) | 67.0 (62.5; 71.7) | 66.3 (61.8; 71.1) | 0.092 |
| **Leafy vegetables** | **9.9 (8.9; 11.0)** | **10.8 (9.8; 11.9)** | **12.8 (11.6; 14.1)** | **<0.001** |
| Fruiting vegetables | 79.1 (74.8; 83.7) | 82.6 (78.4; 87.1) | 85.7 (81.2; 90.4) | 0.155 |
| **Root vegetables** | **18.1 (17; 19.4)** | **20.5 (19.2; 21.8)** | **22.1 (20.7; 23.6)** | **<0.001** |
| Cabbage | 23.2 (22.0; 24.5) | 22.7 (21.6; 23.9) | 24.3 (23.0; 25.6) | 0.272 |
| Other vegetables | 37.3 (35.6; 39.0) | 35.9 (34.3; 37.4) | 37.4 (35.8; 39.2) | 0.796 |
| Legumes | 1.92 (1.75; 2.11) | 1.92 (1.76; 2.10) | 2.03 (1.85; 2.22) | 0.297 |
| **Fruits** | **161.9 (150.1; 174.5)** | **188.5 (175.6; 202.4)** | **215.7 (200.6; 232.0)** | **<0.001** |
| **Nuts and seeds** | **1.50 (1.32; 1.70)** | **2.25 (1.99; 2.54)** | **2.52 (2.23; 2.85)** | **<0.001** |
| Milk | 35.7 (29.2; 43.8) | 39.4 (32.5; 47.8) | 38.7 (31.8; 47.2) | 0.494 |
| **Dairy products** | **74.0 (67.8; 80.7)** | **91.3 (84.1; 99.1)** | **90.5 (83.2; 98.4)** | **0.045** |
| **Cheese** | **29.7 (28.0; 31.6)** | **32.4 (30.6; 34.3)** | **32.9 (31.0; 34.9)** | **0.027** |
| **Bread** | **98.6 (91.6; 106.1)** | **91.6 (85.5; 98.3)** | **86.7 (80.7; 93.2)** | **0.001** |
| Pasta and rice | 26.2 (23.9; 28.8) | 26.2 (24.0; 28.7) | 27.6 (25.2; 30.3) | 0.207 |
| **Other cereals (flour, flakes, starches, semolina, dough and pastry, breakfast cereals)** | **9.8 (9.2; 10.5)** | **11.3 (10.6; 12.0)** | **11.0 (10.3; 11.7)** | **0.016** |
| Beef | 11.0 (9.7; 12.5) | 8.8 (7.8; 9.8) | 10.3 (9.2; 11.7) | 0.736 |
| **Pork** | **20.0 (17.1; 23.3)** | **15.2 (13.1; 17.6)** | **13.7 (11.8; 15.9)** | **0.001** |
| **Poultry** | **0.56 (0.49; 0.64)** | **0.58 (0.51; 0.65)** | **0.40 (0.35; 0.45)** | **<0.001** |
| **Processed meat** | **42.9 (40.1; 46.0)** | **38.7 (36.2; 41.3)** | **33.9 (31.7; 36.3)** | **<0.001** |
| Other meat (offals, hash, other meat) | 15.3 (14.1; 16.5) | 15.3 (14.3; 16.5) | 15.1 (14.1; 16.3) | 0.192 |
| **Fish, fish products** | **16.7 (14.8; 18.7)** | **20.2 (18.1; 22.5)** | **20.6 (18.4; 23.1)** | **0.012** |
| Eggs | 13.5 (12.3; 14.8) | 13.6 (12.5; 14.9) | 13.0 (11.9; 14.2) | 0.225 |
| Butter | 6.5 (5.7; 7.5) | 6.9 (6.1; 7.9) | 6.2 (5.5; 7.1) | 0.694 |
| **Margarine** | **7.7 (6.7; 8.8)** | **8.1 (7.1; 9.3)** | **6.3 (5.5; 7.2)** | **<0.001** |
| **Vegetable oils** | **6.8 (6.3; 7.3)** | **7.5 (7.0; 8.0)** | **8.4 (7.9; 9.0)** | **<0.001** |
| **Other fats** | **1.95 (1.86; 2.04)** | **1.77 (1.70; 1.85)** | **1.82 (1.74; 1.90)** | **0.007** |
| Sugar products (e.g. syrups, candy, ice cream, desserts) | 32.9 (30.6; 35.5) | 33.7 (31.5; 36.2) | 30.9 (28.7; 33.2) | 0.107 |
| Chocolate sweets | 9.5 (8.7; 10.3) | 9.2 (8.5; 9.9) | 8.9 (8.2; 9.6) | 0.198 |
| Cake, cookies | 47.5 (43.6; 51.7) | 50.9 (47.0; 55.2) | 43.3 (39.8; 47) | 0.165 |
| **Non-alcoholic beverages** | **771.2 (691.0; 860.7)** | **831.7 (749.7; 922.7)** | **904.5 (813.4; 1005.9)** | **0.014** |
| **Soft drinks** | **35.1 (29.2; 42.2)** | **20.6 (17.3; 24.5)** | **16.1 (13.5; 19.2)** | **<0.001** |
| Coffee | 320.7 (286.1; 359.6) | 361.0 (324.0; 402.2) | 316.1 (283.0; 353.1) | 0.451 |
| **Tea** | **64.2 (52.8; 78.1)** | **89.8 (74.6; 108.0)** | **118.4 (98.0; 143.1)** | **<0.001** |
| Beer | 10.6 (7.8; 14.5) | 11.0 (8.2; 14.8) | 11.8 (8.8; 16.0) | 0.702 |
| **Wine** | **20.8 (17.7; 24.3)** | **31.2 (26.9; 36.3)** | **55.1 (47.3; 64.2)** | **<0.001** |
| Other alcoholic beverages | 2.66 (2.05; 3.45) | 2.51 (1.96; 3.02) | 2.65 (2.06; 3.40) | 0.270 |
| **Sauces** | **47.2 (44.6; 49.9)** | **44.4 (42.1; 46.8)** | **43.9 (41.6; 46.4)** | **0.034** |
| Soups | 20.1 (18.8; 21.4) | 21.2 (19.9; 22.5) | 20.6 (19.4; 22.0) | 0.254 |
| Bouillon | 14.6 (13.7; 15.7) | 14.7 (13.7; 15.7) | 15.6 (14.6; 16.7) | 0.058 |
| **Soya products** | **1.89 (1.81; 1.98)** | **1.98 (1.90; 2.06)** | **2.12 (2.03; 2.21)** | **<0.001** |
| Dietetic products | 0.25 (0.23; 0.28) | 0.28 (0.26; 0.31) | 0.30 (0.27; 0.33) | 0.081 |
| Snacks | 0.64 (0.58; 0.70) | 0.61 (0.56; 0.67) | 0.57 (0.52; 0.62) | 0.142 |
| **Plant-based diet index** | **68.8 (64.0; 73.9)** | **67.0 (62.5; 71.7)** | **66.3 (61.8; 71.1)** | **<0.001** |
| **Healthy plant-based diet index** | **9.9 (8.9; 11.0)** | **10.8 (9.8; 11.9)** | **12.8 (11.6; 14.1)** | **<0.001** |
| **Unhealthy plant-based diet index** | **79.1 (74.8; 83.7)** | **82.6 (78.4; 87.1)** | **85.7 (81.2; 90.4)** | **<0.001** |

Data are expressed as re-transformed least square means (95% CI) calculated based on ln-transformed food group intakes adjusted for age and sex; **P*_trend_ adjusted for age and sex and calculated based on continuous ln-transformed plasma boron concentrations as dependent variable and ln-transformed food group intakes as independent variable using general linear models.

**Bold** indicates significant associations (*P*<0.05).

Abbreviations: CI, confidence interval; T, tertile.

**Supplemental Table 2**. Power and sample size calculations for the given data for associations of anthropometric and cardio-metabolic variables with plasma boron concentrations

|  | Plasma concentration of boron | |
| --- | --- | --- |
|  | N | Power |
| Age^a^ | 144 | >99.9% |
| Sex^b^ | 19,630 | 9.1% |
| Body mass index^a^ | 657 | 90.8% |
| C-reactive protein^b^ | 1,610 | 55.1% |
| Estimated glomerular filtration rate^a^ | 204 | >99.9% |
| Plasma triglyceride concentration^b^ | 409 | 98.7% |
| Plasma high-density lipoprotein cholesterol concentration^a^ | 1,610 | 55.1% |
| Plasma low-density lipoprotein cholesterol concentration^a^ | 315 | 99.8% |
| Plasma total phosphate concentration^a^ | 134 | >99.9% |
| Education level^b^ | 226 | >99.9% |
| Season^b^ | 3,148 | 31.9% |
| Antihypertensive medication^b^ | 657 | 90.8% |

The table gives participant numbers (n) to detect significant differences based on observed partial correlations for α=0.05 and a power of 80% and calculated power of observed partial correlations based on a participant number of n=899. Models were adjusted for all other covariates identified using stepwise forward selection procedures as indicated in Table 5.

^a^Independent variables not transformed before analysis; ^b^binary variables; boron and ^c^independent variables entered into the models as ln-transformed variables.
